# 

**Published:** 1785

**Authors:** 


### CATALOGUE.

1. THE Works of *John Fothergill*, M. D. F. R. S. &c. with ſome Account of his Life. By *John Coakley Lettſom.* 4to. *Dilly*, London, 1784.

2. An Account of the Life and Writings of the late *William Hunter*, M. D. F. R. S. and S. A. Phyſician Extraordinary to the Queen, &c. Read at a general meeting of the Society of Phyſi-

Phyficians, of which he was Prefident, and publifhed at their requeft. By *Samuel Foart Simmons*, M. D. F. R. S. &c. 8vo. *Johnfon*, London, 1783.

3. Outlines of the Theory and Practice of Midwifery. By *Alexander Hamilton*, M. D. F. R. S. Edin. Profeffor of Midwifery in the Univerfity, and Member of the Royal College of Surgeons, Edinburgh. 8vo. *Elliot*, Edinburgh, 1784.

4. Experiments on the red and quill Peruvian Bark: with Obfervations on its Hiftory, mode of Operation, and Ufes; and on fome other fubjects connected with the phenomena and doctrines of vegetable aftringents. Being a Differtation which gained the firft prize given by the Harveian Society of Edinburgh for the year 1784. By *Ralph Irving*. 8vo. *Elliot*, Edinburgh, 1785.

5. An Inquiry into the Nature and Caufes of Fever; with a review of the feveral opinions concerning its proximate caufe, as advanced by different authors; and particularly, as delivered from the Practical Chair in the Univerfity of Edinburgh; including fome obfervations on the exiftence of putrefaction in the living body, and the proper method of cure to be purfued in

Fever. By *Caleb Dickinſon*, M. D. 8vo. *Elliot*, Edinburgh, 1785.

6. A Hiſtory of the Practice of Trepanning the Skull, and the after-treatment; with obſervations upon a new method of Cure, illuſtrated by a Caſe. By *Robert Mynors*, Surgeon. 8vo. Birmingham, 1785.

In this pamphlet, we are unjuſtly accuſed of having intentionally omitted a material fact, and perverted the ſenſe of a paragraph, in a caſe ſent us by the author*. His aſſertion, that we refuſed to re-print his paper in the ſucceeding number of the Journal, is true; but he ſhould have added what is equally true, that we offered to notice the omiſſion (which related to the adheſion of the ſcalp to the dura mater, and conſiſted of about ten words) among the errata, and that he rejected this offer. With a ſimilar want of candour, he has re-printed his caſe, not in the form in which it was originally ſent to us, but with moſt of our corrections both of ſtyle and arrangement, and with ſome additions, none of which he has pointed out to his readers. What he ſays in vindication of the originality of his mode of practice†, does not at all tend to

* Vol. V. page 278. † Ibid, p. 440.

alter alter our ſentiments on the ſubject; and his arguments certainly acquire no additional weight from the illiberal obſervations with which they are prefaced.

7. An Addreſs to Parliament on the ſituation of the Navy Surgeons; to which are added, Medical Strictures, appertaining to the health of his Majeſty's ſeamen, addreſſed to the Lords of the Admiralty: with obſervations on ſuſpended animation. By *William Renwick*, Surgeon in the Royal Navy. 8vo. *Law*, London, 1785.

9. A Diſſertation on the Theory and Cure of the Cataract, in which the practice of extraction is ſupported, and that operation, in its preſent improved ſtate is particularly deſcribed. By *Jonathan Wathen*, Surgeon. 8vo. *Dilly*, London, 1785.

9. Account of the late epidemic Ague in the neighbourhood of Bridgenorth, Shropſhire, in 1784. By *William Coley*, Surgeon. 8vo. *Murray*, London, 1785.

10. Fragmenta Chirurgica et Medica. Auctore *Gul. Fordyce*, M. D. Eq. Aur. 8vo. *Cadell*, London, 1785.

11. An Inquiry into the various Theories and Methods of Cure in Apoplexies and Palſies. By

By *B. Chandler*, M. D. et Coll. Reg. Med. Lond. permiſſus. 8vo. Canterbury, 1785.

12. Remarks on the Nature and Treatment of morbid Retentions of Urine. By *Charles Brandon Trye*, Member of the Corporation of Surgeons of London, and Surgeon to the General Infirmary at Glouceſter. 8vo. *Murray*, London, 1784.

13. A conciſe relation of the Effects of an extraordinary Styptic, lately diſcovered: In a ſeries of Letters from ſeveral Gentlemen of the Faculty to *Barth. Ruſpini*, Surgeon Dentiſt. 8vo. *Johnſon*, London, 1785.

14. The Philoſophy of Phyſic, founded on one general and immutable Law of Nature, the neceſſarily relative agency of Elementary Fire. By *T. Dewell*, M. D. Malmeſbury, Wilts. The ſecond edition, reviſed and corrected. 12mo. Marlborough, 1785.

15. Almanacco di Sanita. *i. e.* Almanach of Health for the year 1785. 8vo. Turin, 1785.

16. Medinisch-Chirurgiſche beobachtungen auf ſeinen reiſen durch England und Franckreich. *i. e.* Medical and Chirurgical Obſervations made in the Hoſpitals of England and France. By *J. Hunczouſky*. 8vo. Vienna, 1784.

17. Tra-

17. Tratado Medico-practico del Garotillo maligno ulcerado. *i. e.* Treatise on the ulcerated malignant sore Throat. By Don *Juan Antonio Pasqual y Rubio.* 4to. Valencia, 1784.

The chief design of this work is to recommend the use of the Bark.

18. *Andreæ Bonn,* Descriptio Thesauri Ossium morbosorum Hoviani; adnexa est Dissertatio de Callo. 4to. Amsterdam.

19. De Infanticidio non temere admittendo. Auctore *Christ. Godef. Gruner,* Med. Doct. et Prof. 8vo. Jena, 1784.

20. *Barthol. Eustachii* anatomici summi, Romanæ archetypæ Tabulæ anatomicæ, novis explicationibus illustratæ ab *Andr. Maximino.* Folio. Romæ, 1783.

21 *Jo. Andreæ Murray,* M. D. Eq. ord. R. de Wasa, &c. Opuscula, in quibus Commentationes varias tam medicas quam ad rem naturalem spectantes retractavit, emendavit, auxit, cum figuris æneis. Vol. I. 8vo. Gottingen, 1785.

22. *Joannis Weisz,* M. D. Pyretologiæ practicæ tentamen. Editio secunda. 8vo. Viennæ, 1783.

23. *Joannis Weisz,* M. D. Continuatio prima tentaminis Pyretologiæ practicæ, sistens febres cardinales cardinales primas inflammatorias. 8vo. Viennæ, 1783.

24. Confiderations fur le magnetifme animal, ou fur la theorie du monde et des êtres organifés, d'aprés les principes de M. Mefmer, &c. *i. e.* Thoughts on Animal Magnetifm, or on the Theory of the Earth, and of organifed Bodies, on the principles of M. Mefmer. By *M. Bergaffe.* To which are added, Remarks on Motion, by the Marquis *de Chateleux*, of the French Academy. 8vo. Hague, 1784.

25. Differtatio botanico-medica de quibufdam plantis Belgicis in locum exoticarum fufficiendis, &c. Auctore *P. E. Wauters*, Med. Gandavi. 8vo. Ghent, 1785.

26. Tratado de las epidemias malignas y enfermedades particulares de los exercitos, con advertencias à fus generales, ingenieros, medicos, y cirujanos. Nueva maquina ventilatoria, &c. Por le Doctor *Francifco Bruno Fernandez*, Prefbitero, &c. *i. e.* Treatife on the malignant epidemics and difeafes peculiar to armies, with hints to general officers, engineers, phyficians, and furgeons. With a defcription of a new ventilating machine, &c. By Doctor *Francifco Bruno Fernandez*, Prieft, &c. 4to. Madrid, 1785.

